# *Morbillivirus*-host interaction: lessons from aquatic mammals

**DOI:** 10.3389/fmicb.2012.00431

**Published:** 2012-12-24

**Authors:** Giovanni Di Guardo

**Affiliations:** Department of Comparative Biomedical Sciences, Faculty of Veterinary Medicine, University of TeramoTeramo, Italy

In the last 25 years, no less than 10 dramatic morbilliviral epidemics have occurred among free-ranging pinniped and cetacean species and populations worldwide. The origin(s) of the new *Morbillivirus* genus members causing these mass mortality events, along with the reason(s) behind their “sudden” appearance among wild aquatic mammals, are totally unknown (Di Guardo et al., 2005, 2011b).

Interestingly, several cases of chronic infection by *Dolphin Morbillivirus* (DMV) sharing striking features in common with human *Measles Virus* (MeV)-related “*Subacute Sclerosing Panencephalitis*” (SSPE) (Rima and Duprex, 2006), as well as with *Canine Distemper Virus* (CDV)-related “*Old Dog Encephalitis*” (ODE) (Rima et al., 1987), have been recently described in Mediterranean striped dolphins (*Stenella coeruleoalba*) and bottlenose dolphins (*Tursiops truncatus*). Indeed, these DMV-infected cetaceans showed immunohistochemical and/or biomolecular evidence of morbilliviral antigen and/or genome exclusively in their brain tissue (Di Guardo et al., 2011a, 2012; Soto et al., 2011), similarly to what reported in SSPE-affected patients (Rima and Duprex, 2006) and ODE-affected dogs (Rima et al., 1987).

Apart from being highly pathogenic for their hosts, morbilliviruses are lymphotropic as well as epitheliotropic and neurotropic agents (Sato et al., 2012). In this respect, the key determinant of *Morbillivirus* lymphotropism is a cell receptor called “*Signaling Lymphocyte Activation Molecule*” (SLAM), or CD150, a high-affinity ligand for morbilliviral hemagglutinin (H) which is expressed on human and murine thymocytes, activated lymphocytes, mature dendritic cells, macrophages, and platelets (Sato et al., 2012). Likewise, nectin-4—a cell adhesion molecule—has been recently identified as an additional receptor selectively binding MeV H antigen, thereby allowing viral entry into host's epithelial cells (Sato et al., 2012).

Intriguingly enough, both SLAM and nectin-4 are not expressed by neuronal and endothelial cells (Sato et al., 2012), whereas morbilliviruses—which are known to be carried by SLAM^+^ immune cells into the bloodstream (Sato et al., 2012)—necessarily need to cross the blood-brain barrier before invading the host's cerebral parenchyma with its resident neuron cell populations. Therefore, how does the virus achieve these “ambitious” goals and, not less importantly, how does it persist “undisturbed” inside the host's brain, thus progressively giving rise to human SSPE (Rima and Duprex, 2006), canine ODE (Rima et al., 1987), and their “disease analogue” in wild dolphins (Di Guardo et al., 2011a, 2012; Soto et al., 2011)? One possible answer to the above questions could reside in a “selectively/exclusively neurotropic behavior” of given morbilliviral strains inside their hosts, with a crucial determinant of such a behavior being likely represented by the specific interaction of the virus with a receptor molecule selectively and/or consistently expressed by neurons. In this context, CD147—a transmembrane glycoprotein and a gamma-secretase subunit—might be an interesting “candidate molecule,” being expressed by a number of cell types including brain neuronal and capillary endothelial cells (Nahalkova et al., 2010). Indeed, CD147 is a specific ligand for both cyclophilin A (CypA) and CypB, the latter having been also shown to be selectively incorporated into MeV surface, thereby allowing viral particles to interact with the CD147 cell receptor independently of MeV H antigen (Sato et al., 2012).

Could also CDV in dogs and DMV in dolphins “benefit” from a similar pathogenetic mechanism, which should be regarded as complementary/alternative to H antigen interaction with the other hitherto characterized MeV-specific receptors, including CD46, SLAM, nectin-4, and heparin-like glycosaminoglycans (Sato et al., 2012)? And, if so, could CypB incorporation into the viral surface contribute, to some extent, to *Morbillivirus* prolonged persistence inside the host's brain, with subsequent development of human SSPE, canine ODE, and SSPE/ODE-like cases of morbilliviral infection in dolphins? In this respect, while a number of virus-specific and host immune response-related factors likely play a prominent role in the pathogenesis of the aforementioned neurologic disease conditions (Reuter and Schneider-Schaulies, 2010), it should be also emphasized that a significant reduction of tumor necrosis-alfa (TNF-alfa) expression on behalf of macrophages has been recently documented following exposure of these cells to CypB (Marcant et al., 2012). Being absolutely plausible and conceivable that a similar CypB-induced reduction of TNF-alfa expression could also affect microglia, the macrophage-like innate immune cells of the central nervous system (CNS), this would provide the CypB-coated virus with an “extra-capability” of escaping the host's immune response, the efficiency of which is already less consistent, under physiological conditions, at the CNS level (Reuter and Schneider-Schaulies, 2010). Furthermore, both MeV and CDV, along with the more recently characterized *Phocine Distemper Virus* (PDV), have been shown to undergo point- and hypermutation events involving virus envelope genes, such as those coding for fusion (or F protein) and matrix (or M protein), a polypeptide which is needed for viral budding and which has been also implicated in the pathogenesis of human SSPE (Reuter and Schneider-Schaulies, 2010; Philip Earle et al., 2011; Buchanan and Bonthius, 2012). In light of the above, it seems therefore more than likely that the aforementioned mutational events should provide a strong biological plausibility for prolonged *Morbillivirus* persistence inside the host's CNS. Additionally, although we do not know if parallel infection modalities take place in the CNS compartment of cetaceans, it would be worthwhile trying to investigate whether a similar pathogenetic mechanism occurs, or not, also in DMV-infected dolphins.

In conclusion, I believe this is an extremely challenging and fascinating area of research, with aquatic mammals in general, and cetaceans in particular, providing useful lessons and valuable “food for thought” as reliable models of comparative neuropathology and viral neuropathogenesis.
